# Career destinations of graduates from a medical school with an 18-week longitudinal integrated clerkship in general practice: a survey of alumni 6 to 8 years after graduation

**DOI:** 10.1007/s11845-020-02260-0

**Published:** 2020-05-27

**Authors:** Liam G. Glynn, Andrew O. Regan, Monica Casey, Peter Hayes, Michael O’Callaghan, Patrick O’Dwyer, Aidan Culhane, John Cuddihy, Billy O. Connell, Gary Stack, Gerry O’Flynn, Patrick O’Donnell, Raymond O’Connor, Helena McKeague, Deirdre Mc Grath

**Affiliations:** 1 Graduate Entry Medical School, University of Limerick, Limerick, Ireland; 2 Health Research Institute, University of Limerick, Limerick, Ireland

**Keywords:** Career choice, General practice, Medical education, Primary health care, Survey

## Abstract

**Background:**

There is a worldwide recruitment and retention crisis in general practice. Workforce planning has identified the need to train more general practitioners as an urgent priority. Exposure of medical students to general practice as part of the formal and hidden curriculum, the use of longitudinal integrated clerkships, and positive experiences and role models in general practice are all thought to be contributing factors to doctors choosing careers in general practice.

**Aim:**

The aim of this study was to identify career destinations of medical school graduates in a medical school with an 18-week longitudinal integrated clerkship in general practice.

**Design and setting:**

This study was conducted in a single graduate entry medical school at the University of Limerick, Ireland.

**Participants:**

Medical school alumni 6–8 years after graduation.

**Method:**

A survey of graduating cohorts of the medical school from 2011 to 2013 was conducted through email and telephone.

**Results:**

There were a total of 175 alumni for the period 2011 to 2013. Data was collected on 92% (161/175) through an online survey, follow-up email and telephone interview, and was triangulated with searches of professional registration databases and information from key informants. Between 6 and 8 years after graduation, a total of 43% of alumni were engaged in general practice as a career.

**Conclusion:**

The reform of the delivery of general practice within medical school curricula should be considered by medical schools, curriculum designers and policy-makers as part of an overall strategy to address the recruitment and retention of general practitioners as part of the global healthcare workforce.

## Introduction

There is a worldwide recruitment crisis in general practice. Workforce planning has identified the need to train more doctors as GPs as an urgent priority. General practice as a career choice and destination for medical school graduates appears to be declining in popularity worldwide [1–3]. Increased workload, lower autonomy and increased regulation are all cited as reasons for this trend which is becoming an issue of crucial importance in medical workforce planning globally [4]. Healthcare systems that have primary care physicians performing a “gatekeeping” role between primary and secondary care have been shown to deliver higher quality care and better patient outcomes at a lower cost [5]. In addition greater primary care physician supply is associated with lower mortality, but per capita supply decreased in the USA between 2005 and 2015 [6]. In the same study in the USA, every 10 additional primary care physicians per 100,000 population was associated with a significant increase in life expectancy versus the same increase in number of specialist physicians. If current trends continue and decreasing numbers of medical school graduates enter higher training in general practice, then the problem of recruitment and retention of general practitioners is likely to worsen. This will have a detrimental impact on healthcare systems in terms of both quality [7] and cost [8].

The recently published *F2 Career Destination Survey* from the UK Foundation Programme Office shows that in 2012, 24.2% of the overall numbers of junior doctors starting specialty training opted for general practice training in 2012. There was large variation noted between medical schools in the proportion of graduates selecting general practice as their specialty training from 11.2% at Cambridge University to as high as 38.5% at Keele University [9]. Why is it then that some medical schools produce more general practitioners than others? A study of graduates from Swiss medical schools reported that undergraduate placement was the most powerful factor influencing a career choice in general practice [10]. In addition, exposure to general practice as part of the formal curriculum, the hidden curriculum and positive experiences and role models in general practice have all been shown to be contributing factors in young doctors choosing to undertake higher training in general practice [10, 11]. Conversely, it has also been reported that medical students can develop negative perceptions of primary care careers due to some of these same factors [4, 12]. The top five medical schools in the UK producing general practitioners include three of the newest, founded under the last UK government, namely, Keele University, the University of East Anglia and Hull York Medical School. All of these three schools have general practice as a key component of their undergraduate curricula and utilize longitudinal integrated clerkships to deliver the general practice component of the curriculum [9]. According to the agreed international definition as determined by the Consortium of Longitudinal Integrated Clerkships [13], a longitudinal integrated clerkship must have the following common core elements:

1. Medical students participate in the comprehensive care of patients over time.
2. Medical students have continuing learning relationships with these patients’ clinicians.
3. Medical students meet, through these experiences, the majority of the year’s core clinical competencies across multiple disciplines simultaneously.

This form of curriculum delivery is based around the principle of “continuity”: continuity of supervision, continuity of curriculum, and continuity of context [14]. This continuity facilitates the student gradually taking on increased responsibility over the duration of the placement—a factor that is instrumental in the development of professional identity [15]. Longitudinal integrated clerkships have been shown to be at least equivalent to traditional block rotations, internationally, in terms of examination results [16]. There is some evidence that longitudinal integrated clerkships in general practice/family medicine appear to promote and maintain primary care career interest in medical students during and after medical school [12]. Previous research on career choices of medical school graduates has been limited by reporting of current training or future career aspirations in isolation [9] as opposed to actual career destinations. Therefore, the aim of the current study was to ascertain the actual career destinations of graduates 6–8 years after graduation from a medical school with an 18-week longitudinal integrated clerkship in general practice.

## Method

### Setting

This study was conducted in the University of Limerick Graduate Entry Medical School (GEMS), located in the Mid-West of Ireland. This is the newest medical school in Ireland and was commissioned in response to the Buttimer report [17], specifically to address among other things the deficiencies in the general practice workforce. The medical school runs a 4-year graduate entry programme and graduated its first cohort of students in 2011, a class of just 32 students, and has now reached a steady state of approximately 150 students per year as part of a 4-year graduate entry medical school curriculum. The rationale for reporting data at least 6 years after graduation was that a minimum of one pre-registration year and 4 years of higher training is required after graduation to begin a career as a GP in Ireland.

Clinical training takes place in years three and four, where students rotate through the major clinical disciplines in affiliated general practice and hospital sites. The curriculum has three main modules or domains: Knowledge of Health and Illness, Clinical and Anatomical Skills, and Professional Competencies. These run concurrently and underpin all learning across the 4 years. They are designed to ensure that all aspects of the skills required to be a doctor are addressed, from the sciences underpinning a rational approach to diagnosis and management, to an awareness of the importance of personal development. In year three, all students undertake a longitudinal integrated clerkship in general practice and primary care of 18-week duration which accounts for 25% of their clinical training. The longitudinal integrated clerkship in general practice takes place at one of 140 teaching practices that form the University of Limerick General Practice Education and Research (ULEARN-GP) network and is consistently rated highly by students in feedback using the Manchester Clinical Placement Index [18].

### Design

A survey of three graduating cohorts from the medical school (2011, 2012 and 2013) was conducted using an online platform [19]; the link for which was sent by email. For non-respondents, a follow-up email and offer of a telephone interview was sent. Data collected was triangulated with searches of professional registration databases from likely countries of career destination (see Table 1).

**Table 1:** Professional registration databases

Ireland	
Medical Council Registrations	https://www.medicalcouncil.ie/Public-Information/Check-the-Register/
Google	https://www.google.ie/
USA	
Doc Info	https://www.docinfo.org/#!/search/query
Medline Plus	https://medlineplus.gov/directories.html
Doctor Finder	https://doctorfinder.ama-assn.org/doctorfinder/
Canada	
Royal College of Physicians & Surgeons Ontario	https://www.cpso.on.ca/Public-Information-Services/Find-a-Doctor
Royal College British Columbia	https://www.cpsbc.ca/physician_search
Royal College Manitoba (Uploaded to site as weekly reports)	http://cpsm.mb.ca/cjj39alckF30a/wp-content/uploads/weeklyreports/PhysicianDirectory.pdf
Alberta	https://search.cpsa.ca/
Nova Scotia	https://cpsnsphysiciansearch.azurewebsites.net/
Newfoundland	http://www.cpsnl.ca/WEB/CPSNL/PhysicianSearch/Physician_Search.aspx
Saskatchewan	https://cps.sk.ca/imis/
Québec	http://www.cmq.org/bottin/index.aspx?lang=en&a=1
New Brunswick (Can search by specialty)	https://cpsnb.org/en/find-physicians/medical-directory
Prince Edward Island	http://cpspei.ca/public-info/physician-search-2/
New Brunswick (Can search by specialty)	https://cpsnb.org/en/find-physicians/medical-directory
Prince Edward Island	http://cpspei.ca/public-info/physician-search-2/

### Analysis

Summary statistical analysis was conducted using SPSS version 24 and appropriately double-checked.

## Results

### Participants

There were a total of 175 alumni for the period 2011 to 2013. Of these, 70% were EU students and 54% were female (Table 2). Of this total number, data was collected on 92% (161/175) through an online survey, follow-up email and telephone interview, and a search of professional registration databases and information from key informants.

**Table 2:** Baseline description of medical school graduates according to year of graduation

Year of graduation	2011 *N* = 32	2012 *N* = 55	2013 *N* = 88	Total *N* = 175
EU origin, *n* (%)	31 (97%)	41 (75%)	51 (58%)	123 (70%)
Female gender, *n* (%)	14 (44%)	31 (56%)	49 (56%)	94 (54%)
Career data response rate, *n* (%)	31 (97%)	50 (91%)	80 (91%)	161 (92%)

### Career destinations

The most common career destination across the three graduating cohorts 6 and 8 years after graduation was general practice, with a total of 43% engaged in this speciality. This did vary from year to year from 48% in 2011 to 38% in 2013. Medicine (19%) was the next most common career destination followed by psychiatry (9%), surgery (8%), anaesthesia (6%), paediatrics (5%) and obstetrics and gynaecology (4%). Other career choices and breakdown for the different graduation cohorts are described in detail in Table 3.

**Table 3:** Career destination 6–8 years after graduation (all careers) of medical school graduates

			Year of graduation			Total
			2011	2012	2013	
Career destination	General practice	Count	15	24	30	69
		% within year of graduation	48.4%	48.0%	37.5%	42.9%
	Medicine	count	4	10	17	31
		% within year of graduation	12.9%	20.0%	21.3%	19.3%
	Surgery	Count	5	1	7	13
		% within year of graduation	16.1%	2.0%	8.8%	8.1%
	Obstetrics and gynaecology	Count	1	3	3	7
		% within year of graduation	3.2%	6.0%	3.8%	4.3%
	Paediatrics	Count	0	2	6	8
		% within year of graduation	0.0%	4.0%	7.5%	5.0%
	Psychiatry	Count	3	3	8	14
		% within year of graduation	9.7%	6.0%	10.0%	8.7%
	Radiology	Count	0	2	0	2
		% within year of graduation	0.0%	4.0%	0.0%	1.2%
	Anaesthesia	Count	2	1	6	9
		% within year of graduation	6.5%	2.0%	7.5%	5.6%
	Emergency medicine	Count	0	2	0	2
		% within year of graduation	0.0%	4.0%	0.0%	1.2%
	Occupational health	Count	0	1	0	1
		% within year of graduation	0.0%	2.0%	0.0%	0.6%
	Pathology	Count	0	1	3	4
		% within Year of Graduation	0.0%	2.0%	3.8%	2.5%
	Microbiology	Count	1	0	0	1
		% within year of graduation	3.2%	0.0%	0.0%	0.6%
Total		Count	31	50	80	161
		% within year of graduation	100.0%	100.0%	100.0%	100.0%

In terms of general practice as a career, there did not appear to be any gender difference across the three graduating cohorts with identical numbers of males and females working in the speciality. However, it did appear that general practice as a career choice was more popular among non-EU students with 52% choosing it in comparison with 39% of EU students. Table 4 describes the percentage of survey responders in each graduation year working in a career according to gender and country of origin.

**Table 4:** General practice as a career destination for medical school alumni 6–8 years after graduation: percentage of survey responders in each graduation year working in a career in general practice, according to gender and country of origin

Year of graduation	Female % (*n*/*N*)	Male % (*n*/*N*)	Non-EU % (*n*/*N*)	EU % (*n*/*N*)	Total % (*n*/*N*)
2011	54 (7/13)	44 (8/18)	100 (1/1)	47 (14/30)	48 (15/31)
2012	53 (16/30)	40 (8/20)	69 (9/13)	41 (15/37)	48 (24/50)
2013	32 (14/44)	44 (16/36)	44 (16/36)	32 (14/44)	38 (30/80)
Overall total for all cohorts combined	43 (37/87)	43 (32/74)	52 (26/50)	39 (43/111)	43 (69/161)

## Discussion

### Summary

In a single graduate entry medical school with an 18-week longitudinal integrated clerkship in general practice, 43% of graduates are working in general practice 6–8 years after graduation, the highest of any speciality. This study appears to confirm the association between the quantity of clinical general practice teaching at medical school and the later career destination of general practice among alumni.

### Comparison with existing literature

The majority of existing literature in this area reports on aspirations or the training pathways of medical graduates, but not on their actual career destination in terms of a chosen specialty. This study adds to the literature by surveying medical school graduates 6 to 8 years after graduation to examine actual career destinations. The number of graduates choosing a career in general practice in the medical school in this study is comparable with the best performing medical schools in this regard in the UK [9]. In a previous survey of medical students in the medical school under study, 19% had general practice as their preferred career option prior to entering medical school, a figure which rose to 29% during medical school [20]. In a further study of those who had completed their longitudinal integrated clerkship in general practice, 46% reported that they were likely to enter general practice after the experience [21] which is comparable with the actual number (43%) in the current study who subsequently did choose a career in general practice.

In addition, medical students in longitudinal integrated clerkships which involve living and working in rural areas are positively influenced towards primary care and rural career choices [22]. Medical students also reported that the clerkship structure created a dynamic learning environment that helped them to more broadly learn about their patients’ diseases and experiences of illness [23]. In a systematic review of how medical students’ career choices are influenced by their interactions with preceptors, longitudinal integrated clerkships’ duration of placement and continuity, relationships with preceptors have the greatest influence on medical students in pursuing a career in general practice and primary care [24]. It has also been clearly demonstrated, for the first time in the UK, that a statistically significant association exists between the quantity of clinical general practice teaching at medical school and later career destination of general practice [25]. Further research on the benefits of longitudinal integrated clerkships for medical students has reported improved ability to work closely with clinical supervisors, better professionalism and deeper understanding of the health system, all of which are indicators of suitability for postgraduate GP training [26]. The learning and development process that has been described on longitudinal integrated clerkships is important but other factors such as intrinsic personality traits are known to influence career intentions of students on longitudinal integrated clerkships [27]. The growing crisis in general practice workforce with its consequent capacity problems [28] is an obvious challenge to providing longitudinal integrated clerkship placements in this setting but GP tutors are generally positive and enthusiastic about such programmes and perceive the potential benefits on recruitment to general practice as a career [29].

Where medical schools train relatively low numbers of GPs, national training bodies may need to target these specific medical schools to emphasize the attractiveness of primary care and improve students’ exposure to general practice during their medical education. The complex world of funding for medical training may make this difficult, and the implications of this for medical schools and their students must be carefully considered [30].

### Strengths and limitations

Strengths of this study are that it provides original data with a high response rate (92%) regarding career destinations 6 to 8 years after graduation. Additional strengths of this study included the method of data collection and the rigour of follow-up and triangulation as well as the fact that the data was based on their career destination not aspirations or current training. However, the study is limited to a single medical school and only includes 3 years of data.

### Implications for policy and research

There appears to be a connection between the quantity and delivery of general practice within an undergraduate curriculum and the number of future general practitioners a medical school is likely to produce [25]. If medical schools, curriculum designers and policy makers are to address workforce planning in general practice, then the results of the current study need to be taken into account. To address recruitment and retention of general practitioners and arrest the decline in general practice as a career choice for medical school graduates, the development and delivery of bespoke models of medical education within existing medical schools or the development of new medical schools with this particular focus is likely to be a successful strategy. The Scottish governments commissioning of the Scottish Graduate Entry Medicine (ScotGEM) programme utilizes such a strategy specifically to address the recruitment and retention of GPs and generalists in rural Scotland by providing 75% of clinical training in the community setting [31]. To improve recruitment of the next generation of GPs, medical schools must provide more high-quality placements in general practice [25] and expose students to a variety of GP academic role models [4]. Further longitudinal research is required involving a larger sample of medical schools nationally and internationally.

## Conclusions

The reform of the development and delivery of general practice within medical school curricula should be considered by medical schools, curriculum designers and policy-makers as part of an overall strategy to address the recruitment and retention of general practitioners as part of the global healthcare workforce.

## References

[1] Koike S, Matsumoto S, Kodama T (2010). Specialty choice and physicians’ career paths in Japan: an analysis of National Physician Survey data from 1996 to 2006. Health Policy.

[2] López-Roig S, Pastor MÁ, Rodríguez C (2010). The reputation and professional identity of family medicine practice according to medical students: a Spanish case study. Aten Primaria.

[3] Lambert T, Goldacre M (2011). Trends in doctors’ early career choices for general practice in the UK: longitudinal questionnaire surveys. Br J Gen Pract.

[4] Barber S, Brettell R, Perera-Salazar R (2018). UK medical students’ attitudes towards their future careers and general practice: a cross-sectional survey and qualitative analysis of an Oxford cohort. BMC Med Educ.

[5] Starfield B (1994). Is primary care essential?. Lancet.

[6] Basu S (2019). Association of primary care physician supply with population mortality in the United States*,* 2005-2015. JAMA Intern Med.

[7] Pereira Gray DJ, Sidaway-Lee K, White E (2018). Continuity of care with doctors—a matter of life and death? A systematic review of continuity of care and mortality. BMJ Open.

[8] Barker I, Steventon A, Deeny SR (2017). Association between continuity of care in general practice and hospital admissions for ambulatory care sensitive conditions: cross sectional study of routinely collected, person level data. BMJ.

[9] Office, U.K.F.P (2012). National F2 career destination survey.

[10] Studerus L, Ahrens R, Häuptle C, Goeldlin A, Streit S (2018). Optional part-time and longer GP training modules in GP practices associated with more trainees becoming GPs - a cohort study in Switzerland. BMC Fam Pract.

[11] Bethune C, Hansen PA, Deacon D (2007). Family medicine as a career option: how students’ attitudes changed during medical school. Can Fam Physician.

[12] Ford CD, Patel PG, Sierpina VS (2018). Longitudinal continuity learning experiences and primary care career interest: outcomes from an innovative medical school curriculum. J Gen Intern Med.

[13] Worley P, Couper I, Strasser R, The Consortium of Longitudinal Integrated Clerkships (CLIC) Research Collaborative (2016). A typology of longitudinal integrated clerkships. Med Educ.

[14] Hirsh DA, Ogur B, Thibault GE, Cox M (2007). “Continuity” as an organizing principle for clinical education reform. N Engl J Med.

[15] Bates J, Konkin J, Suddards C (2013). Student perceptions of assessment and feedback in longitudinal integrated clerkships. Med Educ.

[16] Latessa R, Beaty N, Royal K (2015). Academic outcomes of a community-based longitudinal integrated clerkships program. Med Teach.

[17] Department of Health and Children (2004) Preparing Ireland’s doctors to meet the health needs of the 21st century (Buttimer Report) [26.3.19]; Available from: https://health.gov.ie/blog/publications/preparing-irelands-doctors-to-meet-the-health-needs-of-the-21st-century-buttimer-report/

[18] Dornan T, Muijtjens A, Graham J (2012). Manchester Clinical Placement Index (MCPI). Conditions for medical students’ learning in hospital and community placements. Adv Health Sci Educ.

[19] Inc. S. (2019) www.surveymonkey.com. [cited 2019 May 10th]

[20] Lane G, Dunne C, English A (2014). General practice career intentions among graduate-entry students: a cross-sectional study at Ireland’s newest medical school. Ir Med J.

[21] O’Donoghue S, McGrath D, Cullen W (2015). How do longitudinal clerkships in general practice/primary care impact on student experience and career intention? A cross-sectional study of student experience. Educ Prim Care.

[22] Walters L, Greenhill J, Richards J (2012). Outcomes of longitudinal integrated clinical placements for students, clinicians and society. Med Educ.

[23] Ogur B, Hirsh D (2009). Learning through longitudinal patient care—narratives from the Harvard Medical School–Cambridge integrated clerkship. Acad Med.

[24] Stagg P, Prideaux D, Greenhill J, Sweet L (2012). Are medical students influenced by preceptors in making career choices, and if so how? A systematic review. Rural Remote Health.

[25] Alberti H, Randles HL, Harding A, McKinley RK (2017). Exposure of undergraduates to authentic GP teaching and subsequent entry to GP training: a quantitative study of UK medical schools. Br J Gen Pract.

[26] Birden H, Barker J, Wilson I (2016). Effectiveness of a rural longitudinal integrated clerkship in preparing medical students for internship. Med Teach.

[27] Eley DS (2015). Personality profiles of rural longitudinal integrated clerkship students who choose family medicine. Fam Med.

[28] Owen K (2019). GP retention in the UK: a worsening crisis. Findings from a cross-sectional survey. BMJ Open.

[29] Bartlett M et al (2019) Dundee’s longitudinal integrated clerkship: drivers, implementation and early evaluation. Educ Prim Care:1–8 10.1080/14739879.2018.1564889 30652938

[30] Deakin N (2013). Where will the GPs of the future come from?. BMJ.

[31] University, o.S.A. Scottish Graduate Entry Medicine (ScotGEM) (2019) [cited 2019 May 13th]; https://www.st-andrews.ac.uk/subjects/medicine/scotgem-mbchb/#30454]

